# *Notes from the Field: Burkholderia pseudomallei* Detected in a Raccoon Carcass Linked to a Multistate Aromatherapy-Associated Melioidosis Outbreak — Texas, 2022

**DOI:** 10.15585/mmwr.mm7150a5

**Published:** 2022-12-16

**Authors:** Julia K. Petras, Mindy G. Elrod, Maureen Ty, Pratistha Adams, Dan Zahner, Adam Adams, M. Worth Calfee, Christine Tomlinson, Shannon Serre, Shawn Ryan, Elise Jakabhazy, Jay E. Gee, Zachary Weiner, William A. Bower, Maria E. Negron, Alex R. Hoffmaster, Heidi Honza

**Affiliations:** ^1^Epidemic Intelligence Service, CDC; ^2^Division of High Consequence Pathogens and Pathology, National Center for Emerging and Zoonotic Infectious Diseases, CDC; ^3^Laboratory Leadership Service, CDC; ^4^Environmental Protection Agency, Dallas, Texas; ^5^Texas Department of State Health Services.

*Burkholderia pseudomallei,* the causative agent of melioidosis, is an environmental gram-negative bacterium endemic in tropical and subtropical regions worldwide. *B. pseudomallei* can infect humans and a wide range of animals through percutaneous inoculation, inhalation, or ingestion (*1*). Melioidosis symptoms are nonspecific and vary widely because *B. pseudomallei* can infect any organ of the body, including the brain. In October 2021, the source of a multistate outbreak of melioidosis that involved four human cases in Georgia, Kansas, Minnesota, and Texas was identified as an aromatherapy room spray imported from India* (*2*).

After the discovery of the aromatherapy spray as the outbreak source, the Texas Department of State Health Services (DSHS) learned that a previously healthy pet raccoon, owned by the family of the Texas patient, had broken a bottle of the implicated aromatherapy spray and walked through the liquid. On April 3, 2021, approximately 2 weeks after this exposure, the raccoon displayed acute neurologic symptoms consistent with neurologic melioidosis^†^ and died from an undetermined cause 3 days later. The carcass was wrapped in a cloth robe and buried on the family’s property. The strain found in the aromatherapy bottle (ATS2021) and linked to the outbreak contained a genetic variant, the bimA_Bm_ allele, which is a virulence factor associated with neurologic melioidosis (*3*).

Environmental suitability modeling studies for *B. pseudomallei* suggest that the soil and climate in parts of Texas are suitable for *B. pseudomallei* (*1*). Because of concerns about establishment of *B. pseudomallei* in soil within a setting where the pathogen is not known to be endemic, and out of an abundance of caution, staff members from Texas DSHS Region 2/3, Environmental Protection Agency (EPA) Region 6, and CDC traveled to the Texas property on April 19, 2022, to determine whether there was evidence of *B. pseudomallei* contamination and to decontaminate the burial site. Thirty-two environmental samples^§^ were collected from the burial site and surrounding area, including soil, tree root fragments, and water from a stream downhill from the site. Soil samples were collected directly above, below, and adjacent to the carcass; 10 radial soil samples were collected at 2-, 4-, and 6-ft (0.6-, 1.2-, and 1.8-m) intervals around the carcass, oriented toward the natural drainage path, down to the stream (Figure). The raccoon carcass was found at a depth of approximately 1 ft (30 cm), and 12 tissue samples were collected during field necropsy.^¶^ After sampling, EPA staff members immediately decontaminated the carcass and excavated soil within a 2-ft (0.6-m) circumference of the carcass in germicidal bleach (8.25% sodium hypochlorite, diluted 1:3 with water) overnight for approximately 15 hours (*4*). All samples were tested for *B. pseudomallei* by polymerase chain reaction (PCR) and cultured by CDC. A portion of four of the 12 tissue samples were formalin-fixed by the Dallas County Health and Human Services Laboratory in Texas and tested for *B. pseudomallei* by immunohistochemistry (IHC) at CDC.

**FIGURE:**
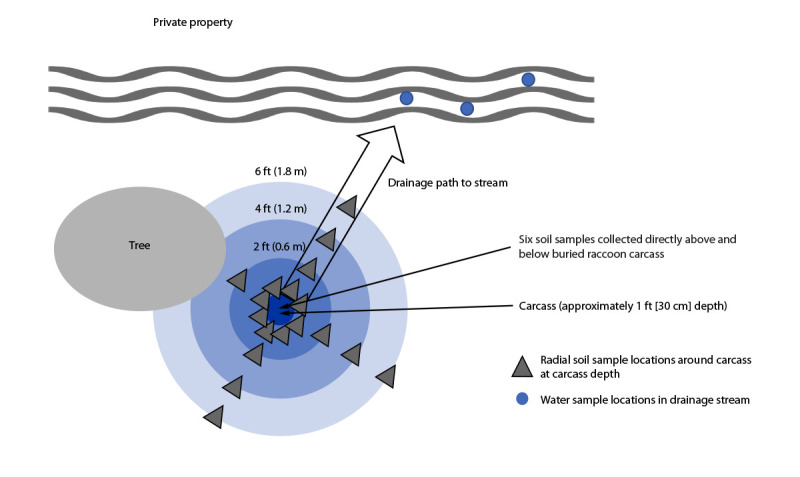
Environmental sample locations around raccoon carcass burial site (aerial view), *Burkholderia pseudomallei* investigation — Texas, 2022

Two swabs collected from the raccoon’s intraorbital tissue tested positive by PCR for the presence of *B. pseudomallei* DNA; however, viable *B. pseudomallei* was not cultured. All other tissue samples tested negative by PCR or IHC.** No environmental contamination was detected, with all environmental samples testing negative for *B. pseudomallei* by both PCR and culture.

The positive PCR result for *B. pseduomallei* from the raccoon tissue reaffirmed the suspicion that the racoon likely died of acute neurological melioidosis. This is the first reported presumed melioidosis case documented in a raccoon and the first animal case linked to this outbreak. Although the bacteria could not be cultured and sequenced, the raccoon was most likely infected by the outbreak strain given the animal’s exposure history and that *B. pseudomallei* has never been isolated from Texas soil. Melioidosis is typically not transmitted from animals to humans; however, it does infect a diverse range of animals including mammals, reptiles, and fish (*1*,*5*). This investigation identified no evidence of environmental contamination by *B. pseudomallei* from the buried carcass; such investigations are important in preventing potential establishment of *B. pseudomallei* in soil within a setting where the pathogen is not known to be endemic.

## Footnotes

*
https://www.cpsc.gov/Recalls/2022/Walmart-Recalls-Better-Homes-and-Gardens-Essential-Oil-Infused-Aromatherapy-Room-Spray-with-Gemstones-Due-to-Rare-and-Dangerous-Bacteria-Bacteria-Identified-in-this-Outbreak-Linked-to-Two-Deaths

† Other considerations in the differential diagnosis included canine distemper and rabies.

§ In addition to the 32 environmental samples collected from the carcass burial site, three control soil samples were collected from public land across the street from the property, which ultimately tested negative; one sample was also collected from the robe in which the carcass was wrapped, which also tested negative.

¶ Tissue samples were taken from right and left orbits (likely brain tissue), intestinal contents, cardiac and pulmonary tissue, intestinal tissue, paw pads, spinal column tissue, adipose and epidermis, and other internal organs that were unidentifiable because of decomposition.

** Ten of the 12 tissue samples tested negative by PCR at the Zoonotic Select Agent Laboratory at CDC, and four tissue samples tested negative by IHC and PCR at the Infectious Diseases Pathology Branch laboratory, CDC.
